# Supplementary material for: Reduced serial dependence suggests deficits in synaptic potentiation in anti-NMDAR encephalitis and schizophrenia
Source: Nat Commun. 2020 Aug 25;11:4250. doi: 10.1038/s41467-020-18033-3 (PMC7447775; doi:10.1038/s41467-020-18033-3)
Supplement: Supplementary file 1 — Supplementary Information [file 41467_2020_18033_MOESM1_ESM.pdf]

Supplementary Material

Reduced serial dependence suggests deficits in synaptic potentiation in anti-NMDAR encephalitis and schizophrenia

Stein, Heike[1,*], Barbosa, Joao[1,*], Rosa-Justicia, Mireia[1,2], Prades, Laia[1], Morató, Alba[1], Galan-Gadea, Adrià[1], Ariño, Helena[1], Martinez-Hernandez, Eugenia[1,3], Castro-Fornieles, Josefina[1,2,4], Dalmau, Josep[1,3,4,5,6], Compte, Albert[1]

[1] IDIBAPS, Barcelona, Spain
[2] Department of Child and Adolescent Psychiatry and Psychology, 2017SGR881, CIBERSAM, Institute Clinic of Neurosciences, Hospital Clínic, Barcelona, Spain
[3] Service of Neurology, Hospital Clínic, Barcelona, Spain
[4] Department of Medicine, University of Barcelona, Barcelona, Spain
[5] Institució Catalana de Recerca i Estudis Avançats (ICREA)-IDIBAPS, Barcelona, Spain
[6] Department of Neurology, University of Pennsylvania, USA

* These authors contributed equally
Corresponding author: acompte@clinic.cat

Supplementary Figures 1-15
Supplementary Tables 1-2

Supplementary Figures

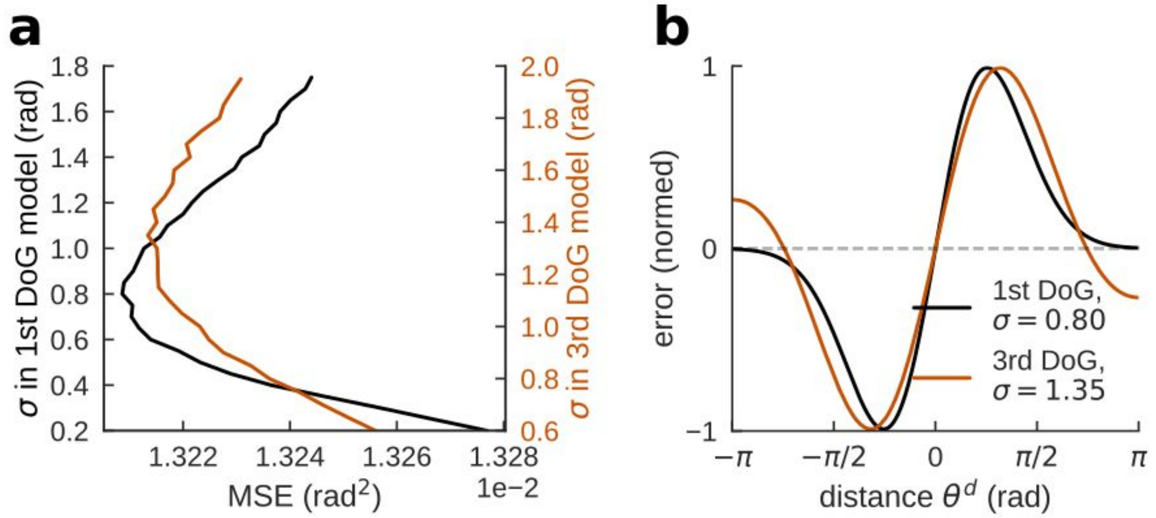

Supplementary Figure 1 | Hyperparameter cross-validation and model selection

a, Mean squared error for stratified hyperparameter optimization using cross-validation (1,000 repetitions, training set size = .33 from each subject) for first- (black) and third- (orange) derivative-of-Gaussian fits. Hyperparameters are different values of scale parameter σ of the underlying Gaussian with location hyperparameter $\mu = 0$. MSE: mean squared error. **b,** Shape of first- and third-derivative-of-Gaussian fits with optimal hyperparameter σ and $\mu = 0$. The cross-validation procedure used for model selection was carried out based on a model with a minimal set of variables (group, delay, and DoG(θ^d)), excluding random effects (Methods, equation (9)). Note that signed previous-current distances in radians were used in the linear model.

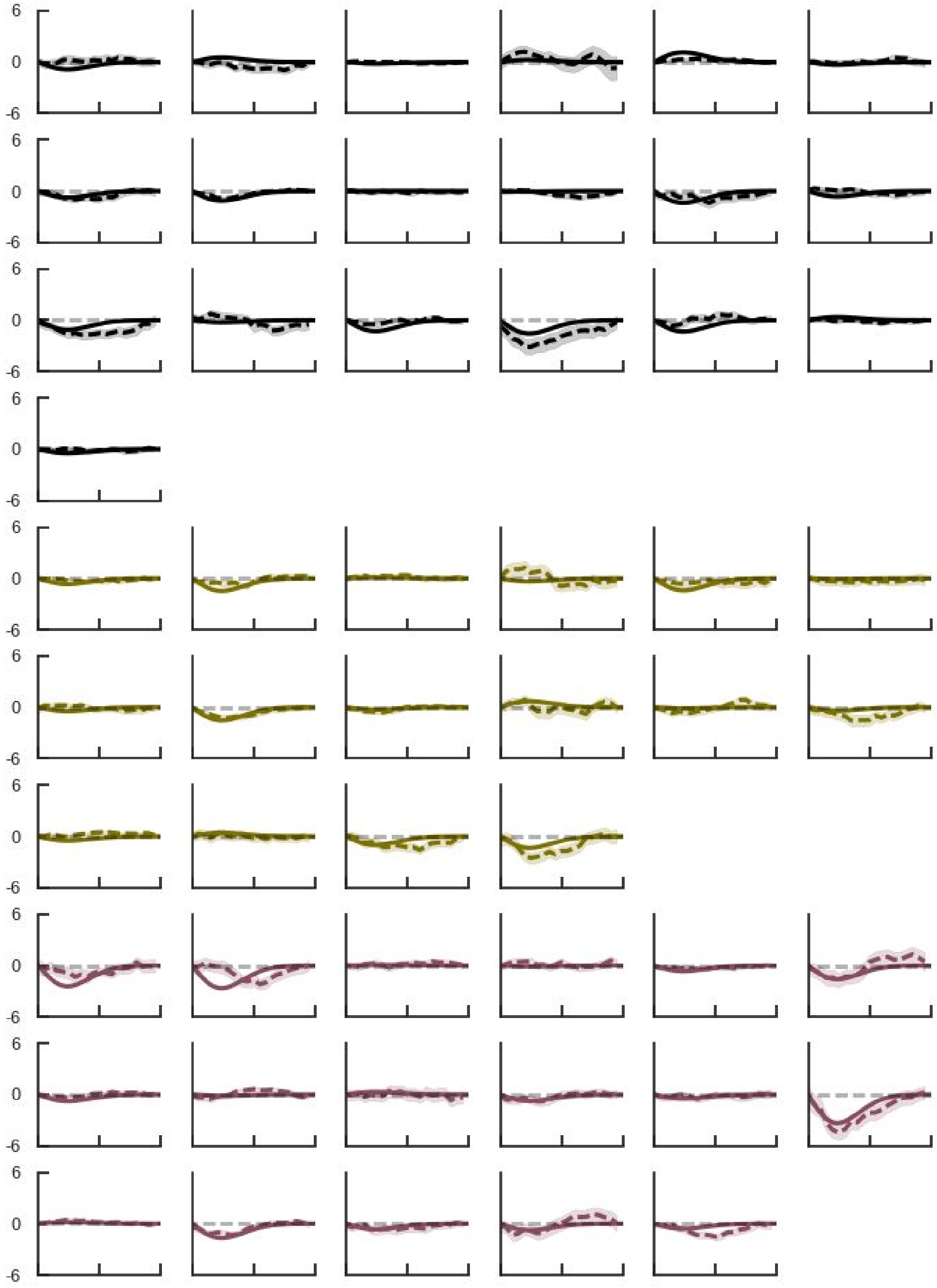

Supplementary Figure 2 | 0 seconds delay single subject bias and linear mixed model fit

Serial dependence is calculated for each subject as the ‘folded’ error θ^{e}’ (in degrees, y-axis) for different previous-current distances θ^{d} (x-axis, spanning absolute values of 0º-180º) (dashed line; Methods). Shading, ± s.e.m. Solid lines show linear model fits (Methods, equation (1)), omitting intercepts and negative values of θ^{d} for visualization. Black curves (row 1-4), ctrl, green curves (row 5-7), enc, purple curves (8-10), schz.

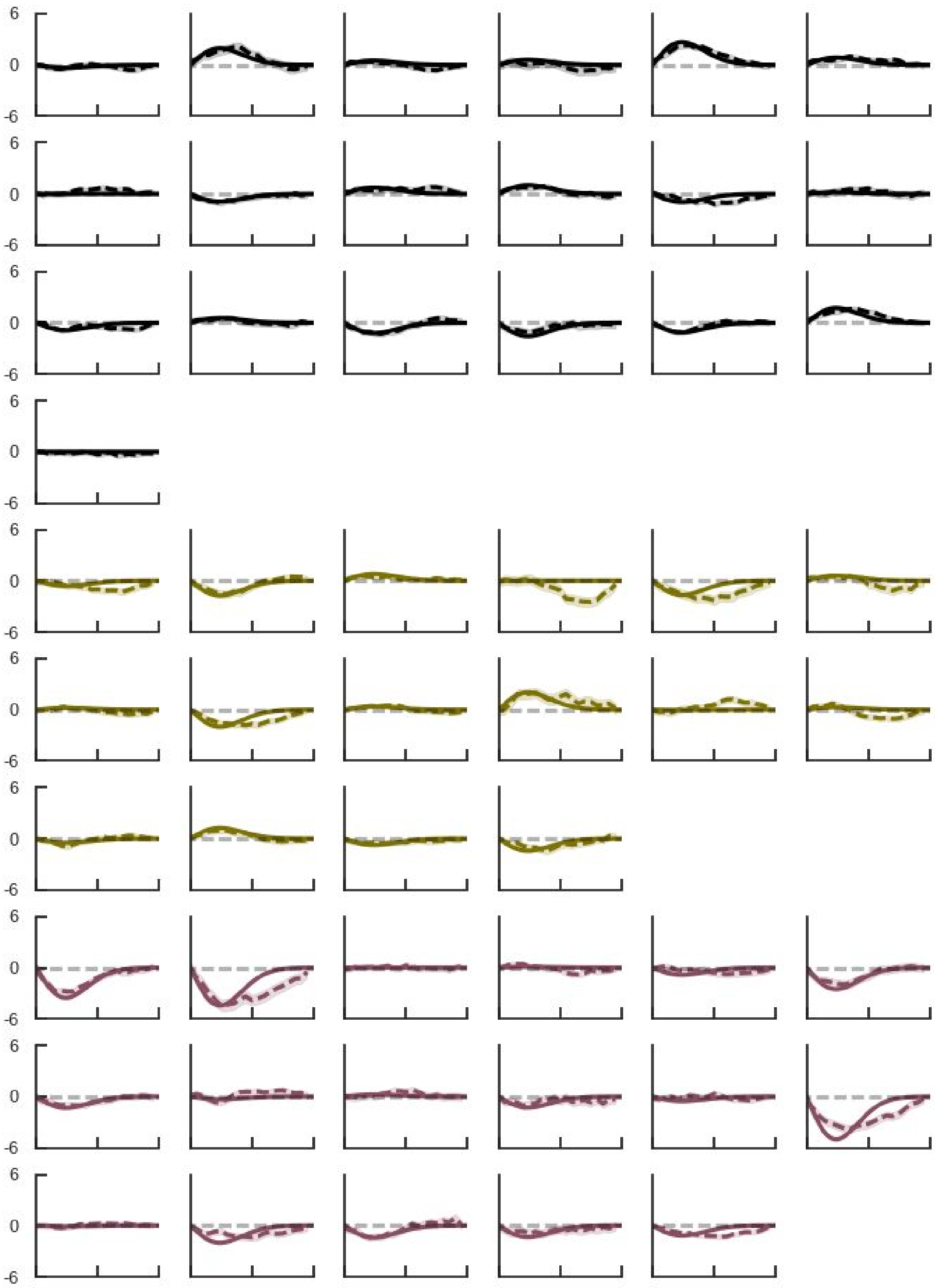

Supplementary Figure 3 | 1 second delay single subject bias and linear mixed model fit

Serial dependence is calculated for each subject as the 'folded' error θ^{e}' (in degrees, y-axis) for different previous-current distances θ^{d} (x-axis, spanning absolute values of 0°-180°) (dashed line; Methods). Shading, ± s.e.m. Solid lines show linear model fits (Methods, equation (1)), omitting intercepts and negative values of θ^{d} for visualization. Black curves (row 1-4), ctrl, green curves (row 5-7), enc, purple curves (8-10), schz.

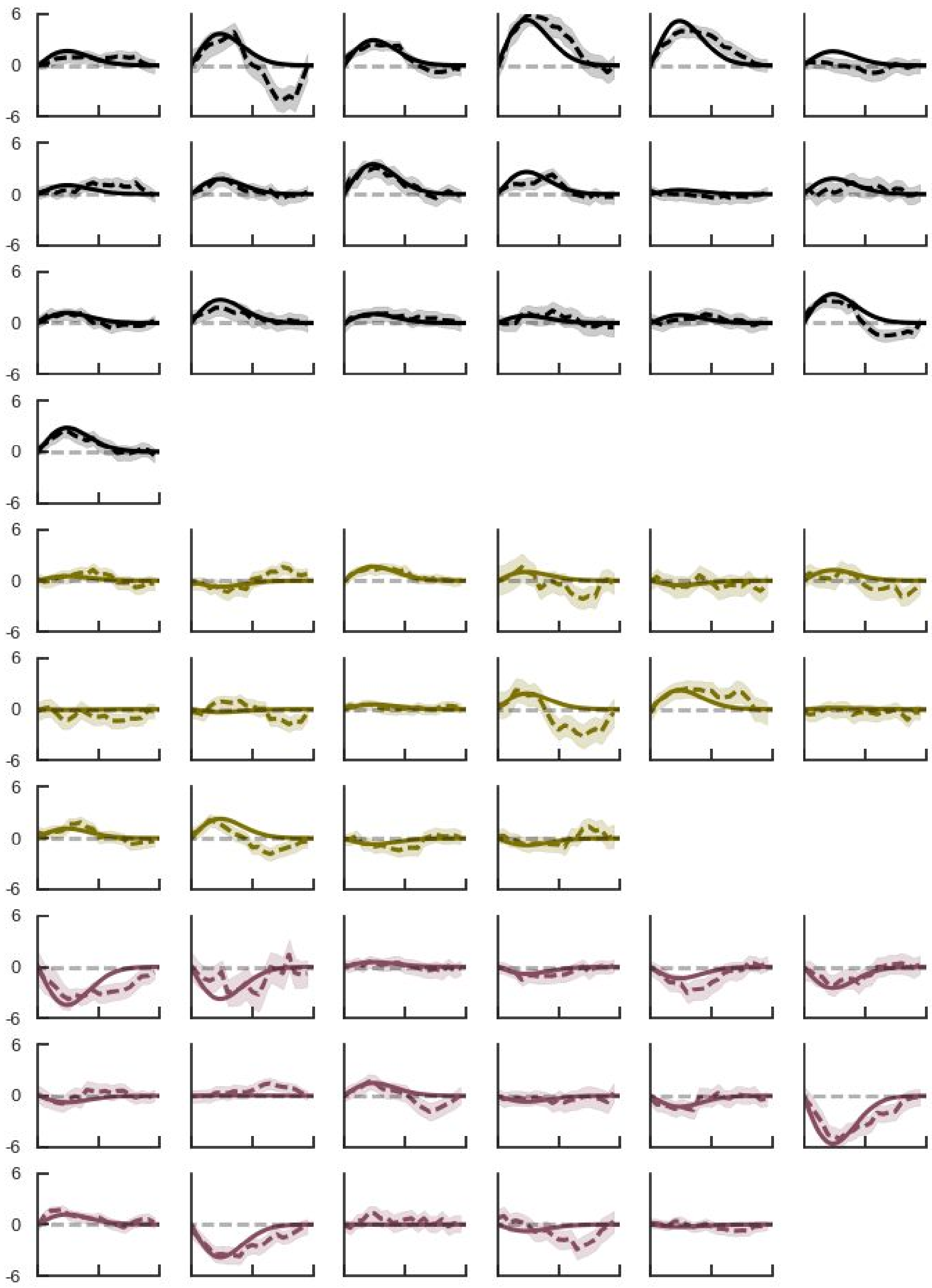

Supplementary Figure 4 | 3 seconds delay single subject bias and linear mixed model fit

Serial dependence is calculated for each subject as the ‘folded’ error θ^{e}’ (in degrees, y-axis) for different previous-current distances θ^{d} (x-axis, spanning absolute values of 0º-180º) (dashed line; Methods). Shading, ± s.e.m. Solid lines show linear model fits (Methods, equation (1)), omitting intercepts and negative values of θ^{d} for visualization. Black curves (row 1-4), ctrl, green curves (row 5-7), enc, purple curves (8-10), schz.

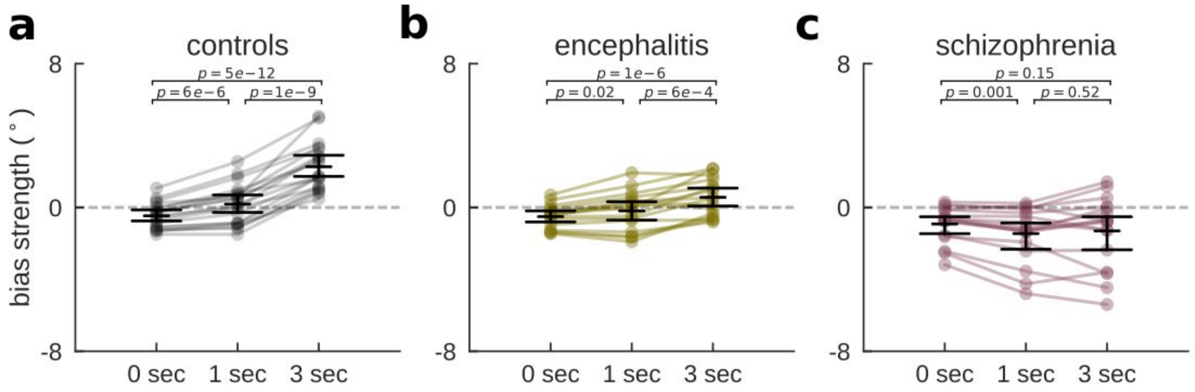

Supplementary Figure 5 | Serial dependence develops as a function of delay length

Individual (random coefficients; dots) and delay-specific group estimates (fixed effects; black horizontal lines indicate mean and bootstrapped 95% C.I. of the mean) of serial dependence. p-values report pairwise comparisons of random coefficients using paired, two-sided t-tests for n = 19 (ctrl), n = 16 (enc), and n = 17 (schz) patients. **a,** Initially repulsive biases became gradually more attractive with delay length for healthy controls (Methods, equation (3); delay × DoG(θ^d), F(2,17) = 26.91, p = 6e-6; 0 vs 1 s: t = -6.33, p = 6e-6, Cohen's d = -1.45; 1 vs 3 s: t = -11.37, p = 1e-9, Cohen's d = -2.6; 0 vs 3 s: t = -15.87, p = 5e-12, Cohen's d = -3.64) and **b,** for encephalitis patients (delay × DoG(θ^d), F(2,23) = 5.06, p = 0.015; 0 vs 1 s: t = -2.71, p = 0.02, Cohen's d = -0.68; 1 vs 3 s: t = -4.32, p = 6e-4, Cohen's d = -1.08; 0 vs 3 s: t = -7.82, p = 1e-6, Cohen's d = -1.95). **c**, schizophrenia patients' biases did not develop over the course of the delay (delay × DoG(θ^d), F(2,16) = 1.31, p = 0.30; 0 vs 1 s: t = 3.99, p = 0.001, Cohen's d = 0.97; 1 vs 3 s: t = -0.65, p = 0.52, Cohen's d = -0.16; 0 vs 3 s: t = 1.53, p = 0.15, Cohen's d = 0.37), but stayed repulsive throughout all delay lengths (DoG(θ^d), F(1,16) = 9.07, p = 0.008).

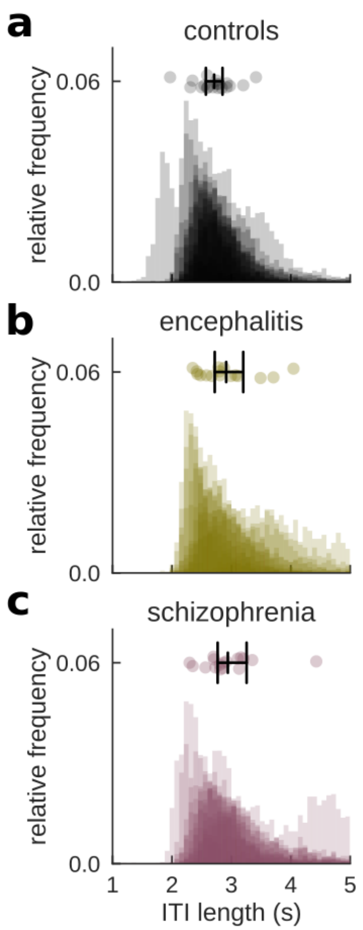

Supplementary Figure 6 | Reduced serial dependence is not explained by group differences in ITI

Histograms of ITI lengths for **a**, control participants **b**, anti-NMDAR encephalitis, and **c**, schizophrenia patients. Here, the ITI is defined as the complete period from probe onset in trial n-1 to stimulus onset in trial n (including previous-trial response). Each plot shows normalized histograms, transparently overlayed for each participant. Points on top show median ITI lengths for each participant (n = 19 healthy controls, n = 16 patients with encephalitis, and n = 17 patients with schizophrenia), together with group mean and bootstrapped 95% C.I. (black middle line and error bars). There was a trend for longer median ITIs in patient groups (Kruskal-Wallis test for median ITI length, H = 5.17, p = 0.08; ctrl, 2.71±0.32 s; enc, 2.91±0.49 s; and schz, 3.03±0.46 s; mean±s.d.). Including ITI × DoG(θ^d) in our linear model (Methods, equation (4); ΔAIC = -13.8) did not change group or delay effects of serial dependence (delay × DoG(θ^d), F(2,58) = 14.03, p = 1e-5; group × DoG(θ^d), F(2,50) = 8.13, p = 9e-4; group × delay × DoG(θ^d), F(4,58) = 8.45, p = 2e-5), but rather explained additional variance (ITI × DoG(θ^d), F(1,7503) = 15.76, p = 7e-5).

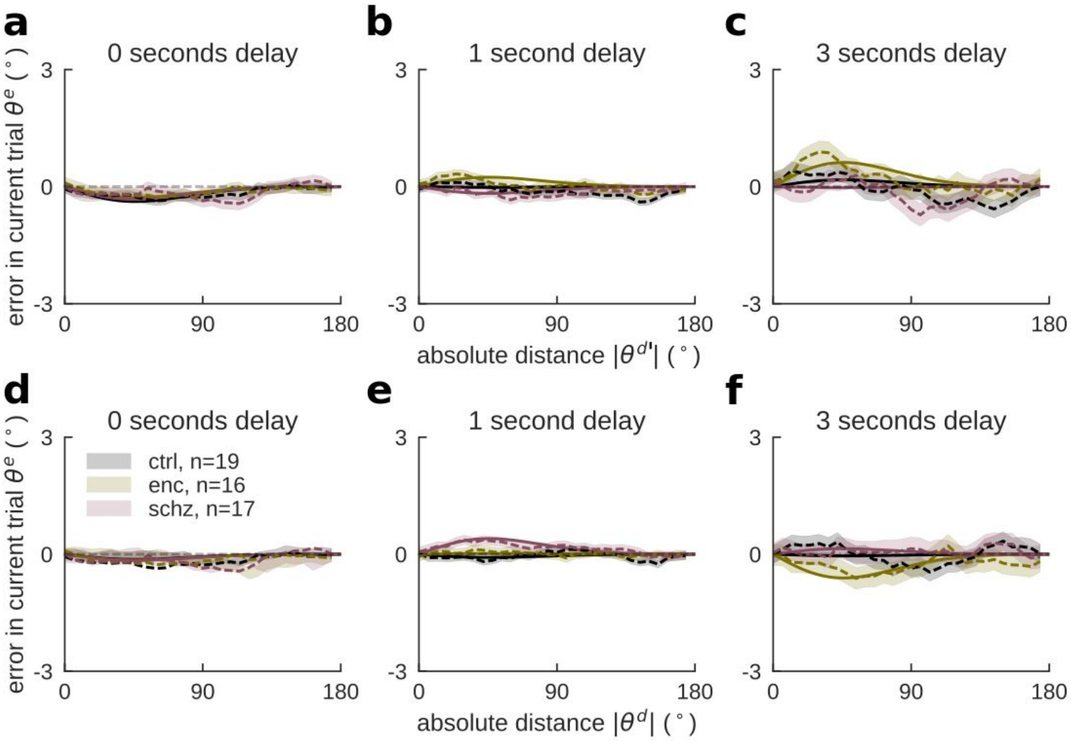

Supplementary Figure 7 | Serial dependence to stimulus n-2 and stimulus n+1

a,b,c, An extended linear model with bias terms to both n-1 and n-2 stimuli (adding the $\theta^{d'}$-dependent term group × delay × DoG($\theta^{d'}$), Methods, equation (7); ΔAIC = -4.11) showed significant delay-dependent bias towards the penultimate stimulus (delay × DoG($\theta^{d'}$), $F(2,52269) = 5.43$, $p=0.004$). Group differences could not be discarded (group × DoG($\theta^{d'}$), $F(2,52276) = 2.86$, $p = 0.06$), but there was no evidence for delay-dependent group differences (group × delay × DoG($\theta^{d'}$), $F(4,52268) = 0.47$, $p = 0.76$). Groupwise models for each delay showed **a,** significant repulsive bias (DoG($\theta^{d'}$), $F(1,8601) = 15.41$, $p = 9e-5$) but no group differences for delays of 0 s, (group × DoG($\theta^{d'}$), $F(2,8601) = 0.10$, $p = 0.91$). **b,** In contrast, groups differed for 1 s delays in absence of overall bias (DoG($\theta^{d'}$), $F(1,34938) = 0.06$, $p = 0.81$; group × DoG($\theta^{d'}$), $F(2,34938) = 3.38$, $p = 0.03$), but **c,** not for 3 s delays (DoG($\theta^{d'}$), $F(1,8669) = 2.57$, $p = 0.11$; group × DoG($\theta^{d'}$), $F(2,8669) = 1.55$, $p = 0.21$). **c, d, e,** We investigated whether serial dependence to stimulus n-1 and group differences in biases could be explained by general response correlations. To detect potential spurious correlations across trials, we replaced previous-current distances (between trial n and trial n-1) in equation (1) with future-current distances (between trial n and trial n+1), as proposed in ref. [1]. There was no significant overall bias towards future stimuli (DoG(θ^{d}), $F(1,53) = 0.63$, $p = 0.43$; delay × DoG(θ^{d}), $F(2,88) = 2.15$, $p = 0.12$; group × DoG(θ^{d}), $F(2,53) = 1.57$, $p = 0.22$; group × delay × DoG(θ^{d}), $F(4,88) = 1.04$, $p = 0.39$), indicating non-significant contributions of general response correlations between trials to the reported group and delay effects of serial dependence. In all panels, dashed lines show 'folded' errors $\theta^{e\prime}$, and solid lines show linear model fits. Shading, ± s.e.m. across pooled trials from n = 19 healthy controls (ctrl), n = 17 patients with schizophrenia (schz), and n = 16 patients with anti-NMDAR encephalitis (enc).

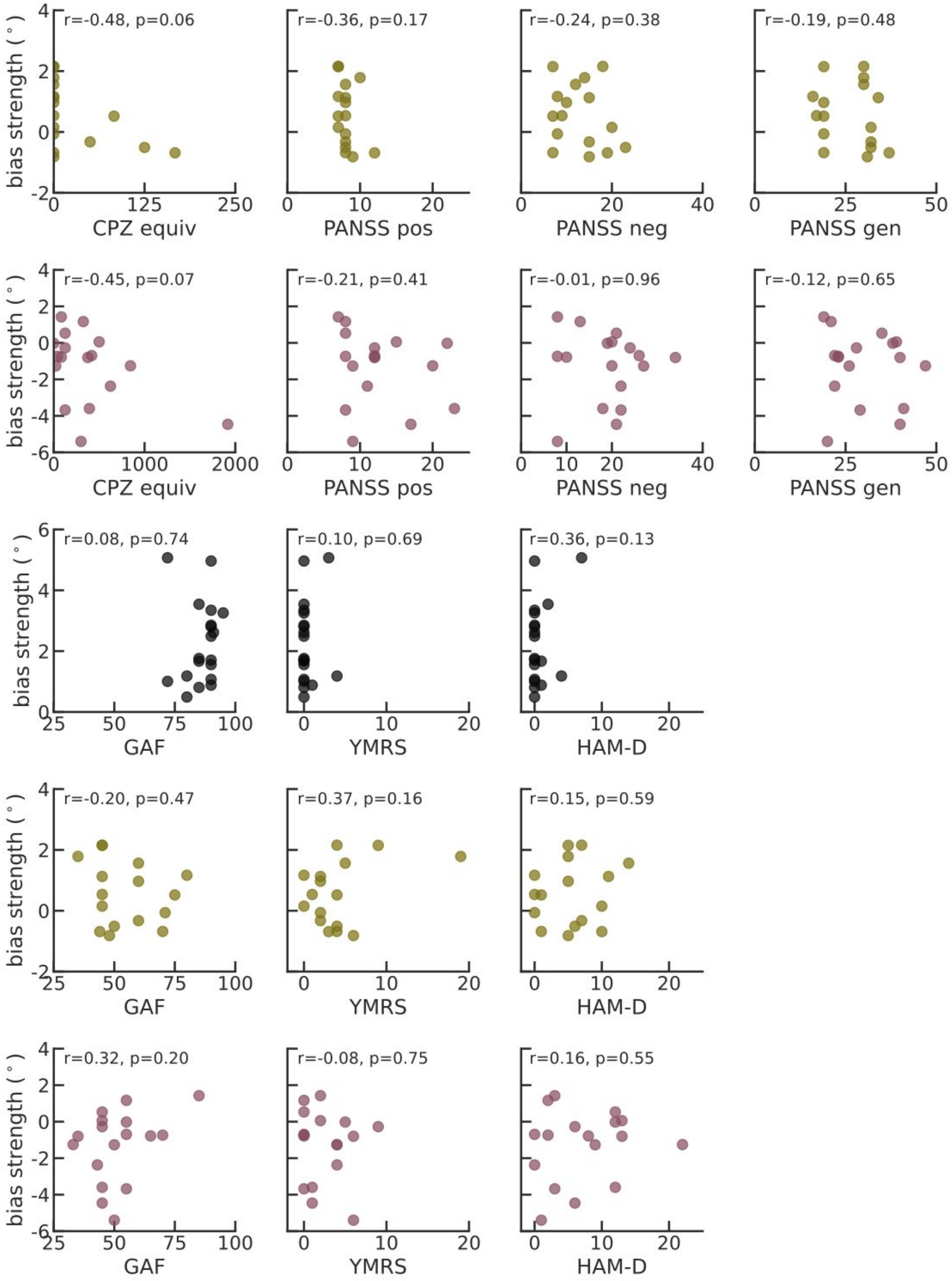
r=-0.48, p=0.06
r=-0.36, p=0.17
r=-0.24, p=0.38
r=-0.19, p=0.48
bias strength (°)
CPZ equiv
PANSS pos
PANSS neg
PANSS gen
r=-0.45, p=0.07
r=-0.21, p=0.41
r=-0.01, p=0.96
r=-0.12, p=0.65
bias strength (°)
CPZ equiv
PANSS pos
PANSS neg
PANSS gen
r=0.08, p=0.74
r=0.10, p=0.69
r=0.36, p=0.13
bias strength (°)
GAF
YMRS
HAM-D
r=-0.20, p=0.47
r=0.37, p=0.16
r=0.15, p=0.59
bias strength (°)
GAF
YMRS
HAM-D
r=0.32, p=0.20
r=-0.08, p=0.75
r=0.16, p=0.55
bias strength (°)
GAF
YMRS
HAM-D

Supplementary Figure 8 | Correlations of serial dependence in 3 seconds delay trials with clinical scales

For each group, we correlated individual bias coefficients for 3 s delay trials (random effects) with clinical measures (Methods for description of administered tests). The strength of serial dependence did not correlate significantly with clinical scales. Correlations were calculated using Pearson's r for n = 19 (ctrl, black, row 3), n = 16 (enc, green, rows 1 and 4), and n = 17 (schz, purple, rows 2 and 5). Correlations with antipsychotic medication (CPZ equivalent) reached marginal significance for both encephalitis and schizophrenia. To test whether medication could account for group differences in delay-dependent bias, we included a transversal estimate of antipsychotic medication, CPZ, as a covariate in our linear model (Methods, equation (5); ΔAIC = -2.7). Antipsychotic medication explained a significant amount of variance in delay-dependent bias (CPZ $\times$ delay $\times$ DoG(θ^d), $F(3,60) = 3.06$, $p = .03$), but did not change the pattern of results (delay $\times$ DoG(θ^d), $F(2,62) = 17.58$, $p = 9e-7$; group $\times$ DoG(θ^d), $F(2,48) = 3.92$, $p = 0.03$; group $\times$ delay $\times$ DoG(θ^d), $F(4,62) = 4.43$, $p = 0.003$). Moreover, to be able to pool all subjects for each of the correlations, we modeled subject-wise bias strength in 3 s trials as a function of group and a second regressor, corresponding to each of the above clinical measures. Models with psychosis-related measures (CPZ and PANSS scales) were fitted on patient data only, and on all subjects' biases for all other models (including GAF, YMRS, HAM-D). In these analyses, only CPZ significantly predicted the strength of serial dependence (CPZ, $F(1,30) = 6.52$, $p = 0.02$), together with group (schz vs enc, $F(1.30) = 4.27$, $p = 0.05$). Measures: GAF (Global Assessment of Functioning Scale [2]), YMRS (Young Mania Rating Scale [3]), HAM-D (Hamilton Depression Rating Scale [4]), PANSS (Positive and Negative Syndrome Scale [5]) Positive, Negative and General Psychopathology Scale, CPZ equivalent (transversal estimate of antipsychotic medication as chlorpromazine equivalent).

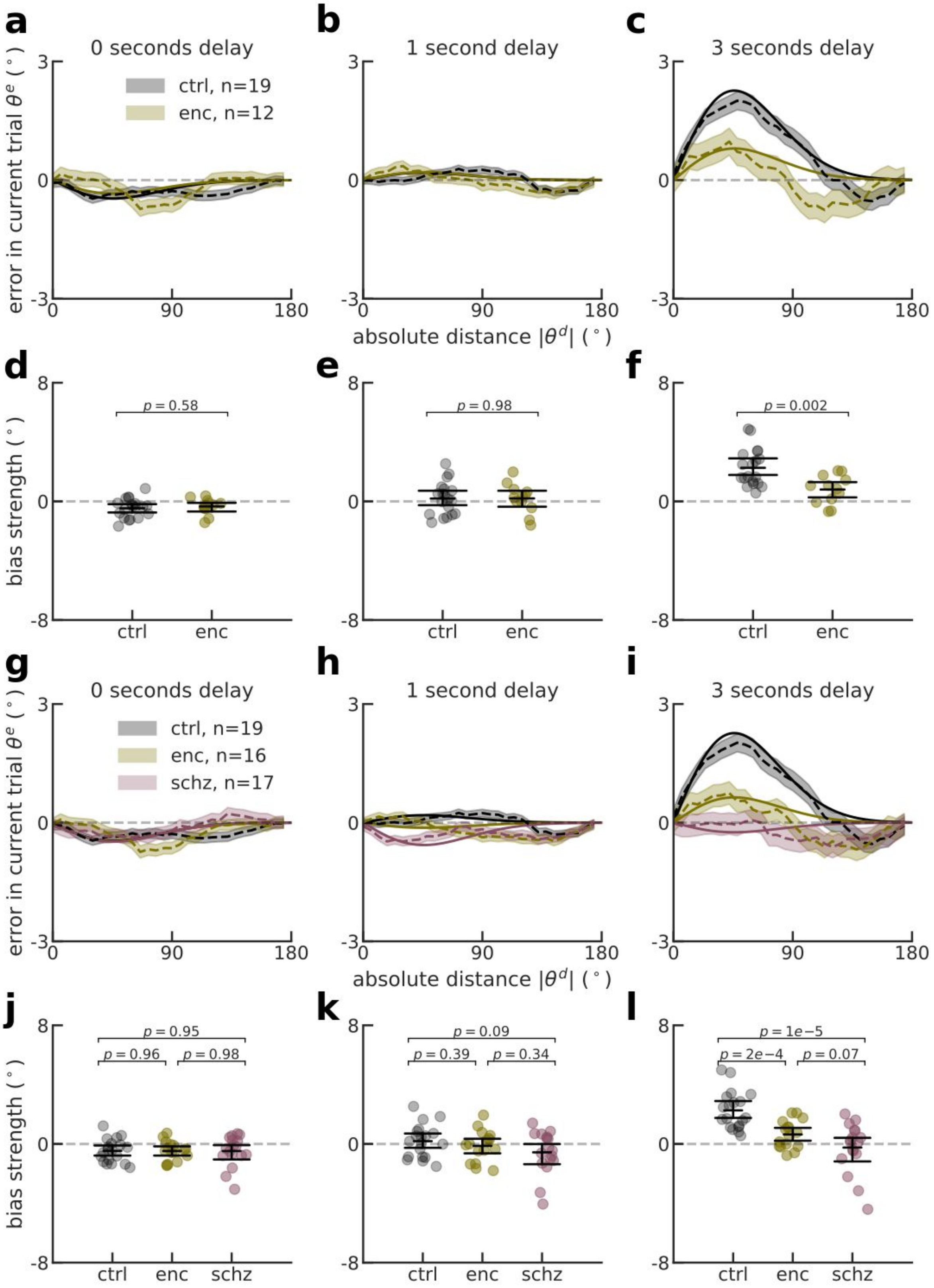

a
b
c
0 seconds delay
1 second delay
3 seconds delay
ctrl, n=19
enc, n=12
error in current trial θ^e ($^\circ$)
absolute distance $|\theta^d|$ ($^\circ$)
d
e
f
bias strength ($^\circ$)
$p = 0.58$
$p = 0.98$
$p = 0.002$
ctrl
enc
g
h
i
0 seconds delay
1 second delay
3 seconds delay
ctrl, n=19
enc, n=16
schz, n=17
j
k
l
$p = 0.95$
$p = 0.96$
$p = 0.98$
$p = 0.09$
$p = 0.39$
$p = 0.34$
$p = 1e{-5}$
$p = 2e{-4}$
$p = 0.07$
ctrl
enc
schz

Supplementary Figure 9 | Pronounced group differences in serial dependence remain after controlling for antipsychotic medication

a-f, To control for potential effects of chlorpromazine equivalent (CPZ, mg day^{-1}) on serial dependence, we fitted our full model as described in equation (1) on the unmedicated subset of participants (ctrl, n = 19 and enc, n = 12 out of n = 16, excluding schz due to n = 1). **a,b,c,** There were significant group differences in delay-dependent biases (group × delay × DoG(θ^d), F(2,28) = 4.49, p = 0.02 with n = 12, as compared to F(2,32) = 5.70, p = 0.008 with n = 16 enc, excluding the schizophrenia group), with no significant overall group differences in biases in neither the subset, nor the full group of patients (group × DoG(θ^d), F(1,29) = 1.29, p = 0.27 for n = 12, and F(1,33) = 3.91, p = 0.06 with n = 16). **d,e,f,** Delay-wise models showed that the group difference in biases occured in 3 s delay trials (F(1,29) = 5.80, p = 0.02 for n = 12), whereas biases were comparable between groups for shorter delays (F(1,30) = 0.15, p = 0.70 for 0 s, and F(1,29) = 0.00, p = 0.99 for 1 s). **g-l,** To obtain a conservative estimate of group differences after removing all possible linear effects of CPZ in all subjects (n=19 healthy controls, n=16 patients with encephalitis, and n=17 patients with schizophrenia), we first regressed trialwise errors on terms containing CPZ (equation (6)), and then estimated group and delay effects (as described in equation (1)) still present in the regression residuals from equation (6). CPZ had a significant effect on delay-independent and delay-dependent biases (CPZ × DoG(θ^d), F(1,52387) = 196.17, p < 1e-16, and CPZ × delay × DoG(θ^d), F(2,52387) = 4.91, p = 0.007. **g,h,i,** Delay-independent and delay-dependent serial biases remained significantly altered in both patient groups after partially regressing CPZ equivalent from errors (group × DoG(θ^d), F(2,49) = 3.54, p = 0.04, and group × delay × DoG(θ^d), F(4,63) = 6.14, p = 0.0003). **j,k,l,** Delay-wise models showed similar results to Figure 1, with equally repulsive serial dependence for all groups in 0 s trials (DoG(θ^d), F(1,52) = 6.76, p = 0.01, and group × DoG(θ^d), F(2,51) = 0.03, p = 0.97) and group differences emerging in 3 s trials (group × DoG(θ^d), F(2,50) = 8.97, p = 0.0005; with significant differences between individual estimates for ctrl vs enc, t = 4.21, p = 2e-4, Cohen's d = 1.47; ctrl vs schz, t = 5.05, p = 1e-5, Cohen's d = 1.74; enc vs schz, t =1.85, p = 0.07, Cohen's d = 0.66), although not in 1 s trials (group × DoG(θ^d), F(2,48.1) = 1.38, p = 0.26). **a-c, g-i,** Dashed lines with ± s.e.m. shading, data; solid lines, linear model fits (Methods). ctrl: healthy controls, schz: schizophrenia, enc: anti-NMDAR encephalitis. **d-f, j-l,** Individual (random coefficients; dots) and group estimates of serial bias strength (fixed effects; black error bars indicate mean and bootstrapped 95% C.I. of the mean) by delay.

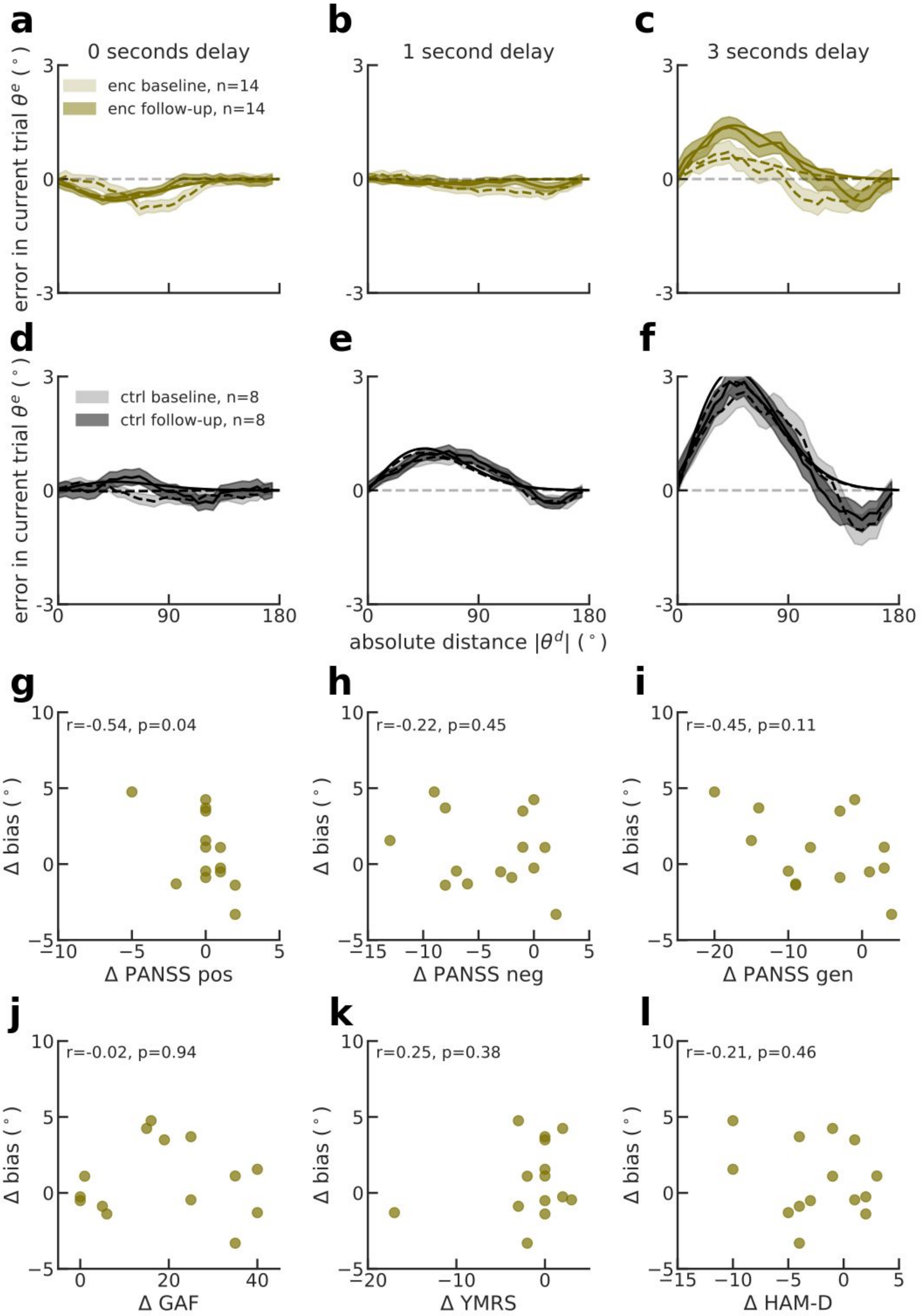

Supplementary Figure 10 | Serial dependence increases with encephalitis patients'

recovery

We performed a comparison of baseline and follow-up sessions (Methods, equation (8)) for n=14 encephalitis patients (enc, Supplementary Table 2) and n=8 controls (ctrl). Although the four-way interaction did not reach significance (session × group × delay × DoG(θ^d), $F(2,30124) = 0.79$, $p = 0.45$), group-wise models showed a normalization of biases in encephalitis patients' (**a-c**, session × delay × DoG(θ^d), $F(2,30124) = 3.07$, $p = 0.046$), and not in healthy controls (**d-f,** $F(2,16311) = 0.10$, $p = 0.90$). A delay-wise comparison of encephalitis patients' baseline and follow-up values showed that this difference was driven by biases in 3 s delays (session × DoG(θ^d), $F(1,5030) = 4.43$, $p = 0.035$), while biases in 0 and 1 s delays did not change ($F(1,5030) = 0.15$, $p = 0.69$, and $F(1,20064) = 0.05$, $p = 0.81$, respectively). Note that due to the increased complexity of the model and the limited sample size, we could not model random effects in this model. In panels a-f, shading denotes mean ± s.e.m. across pooled trials from all subjects of the respective group. **g-l,** To assess single-subject alterations in serial dependence and their correlation with clinical improvement, we estimated subject-wise models in 3 s delay trials for encephalitis patients, by modeling errors θ^e as a function of session, DoG(θ^d), and their interaction, session × DoG(θ^d). We then correlated coefficients for session× DoG(θ^d) (y-axis, Δ bias; positive values denote higher bias in the follow-up session) with change scores in clinical scales (x-axis; positive values denote higher scores in the follow-up session; Pearson's r and uncorrected p-values in panels indicate strength and significance of each correlation). We found that a more accentuated longitudinal reduction in PANSS positive symptoms was related to a stronger increase in memory-dependent biases ($r = -0.54$, C.I. = [-0.83, -0.02], $p = 0.04$).

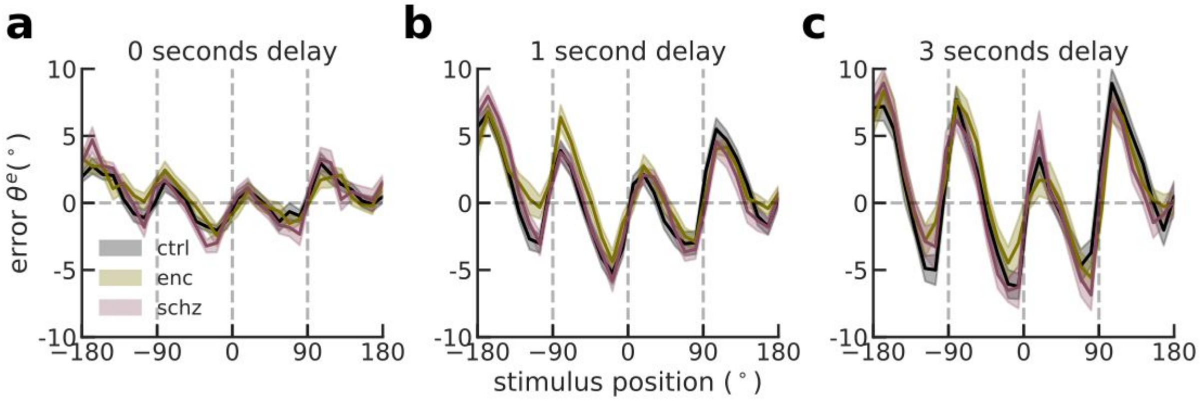

Supplementary Figure 11 | No group differences in working-memory biases with respect to cardinal directions

History-independent response biases, such as attractive or repulsive effects with respect to cardinal directions (0º, ±90º, 180º) could influence behavioral performance and the fidelity of serial dependence estimations. **a,b,c,** Subject-averaged mean trialwise response errors θ^e, binned by stimulus location (x-axis, 30 bins of 12º) for each delay. This analysis revealed the effect of "repulsion from the axes" (e.g. [6–8]) in all groups and delays. Error shading denotes mean ± s.e.m. over n=19 healthy controls (ctrl), n=17 patients with schizophrenia (schz), and n=16 patients with anti-NMDAR encephalitis (enc). To quantify the strength of this effect, we measured the standard deviation (s.d.) of the binned statistic for each subject and delay, and assessed potential group- and delay-differences with an ANOVA. We observed stronger repulsion from the axes with increasing delays (observable in a,b,c; $F(2,147) = 72.45$, $p < 1e\text{-}16$), but no overall group differences ($F(2,147) = 1.72$, $p = 0.18$) or delay-dependent group differences ($F(4,147) = 0.16$, $p = 0.96$).

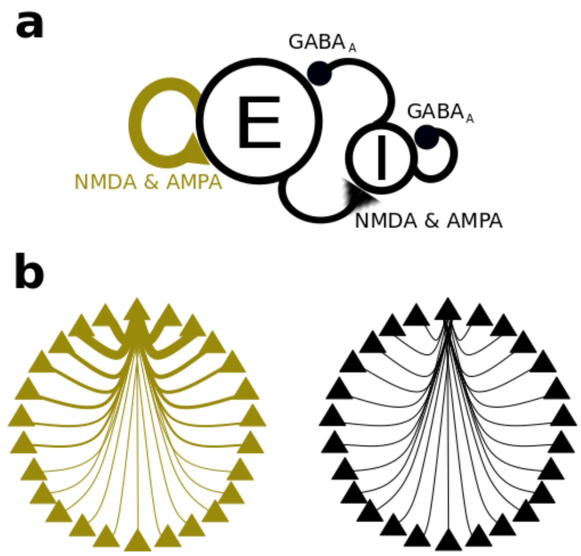

Supplementary Figure 12 | Network scheme and connectivity profile

a, Scheme of spiking neural network, consisting of 1,024 excitatory and 256 inhibitory neurons. Neurons from both pools were connected in an all-to-all fashion, with excitatory connections governed by NMDA and AMPA dynamics, and inhibitory connections governed by $GABA_A$ dynamics. STP affected recurrent excitatory connections. **b,** Weight profiles for recurrent excitatory (green) and all other connections (black). For recurrent connections, weights between neurons preferring similar locations were higher, while more distant neurons were only weakly connected. All other connections had flat connectivity profiles, with equal weights between similar and dissimilar neurons.

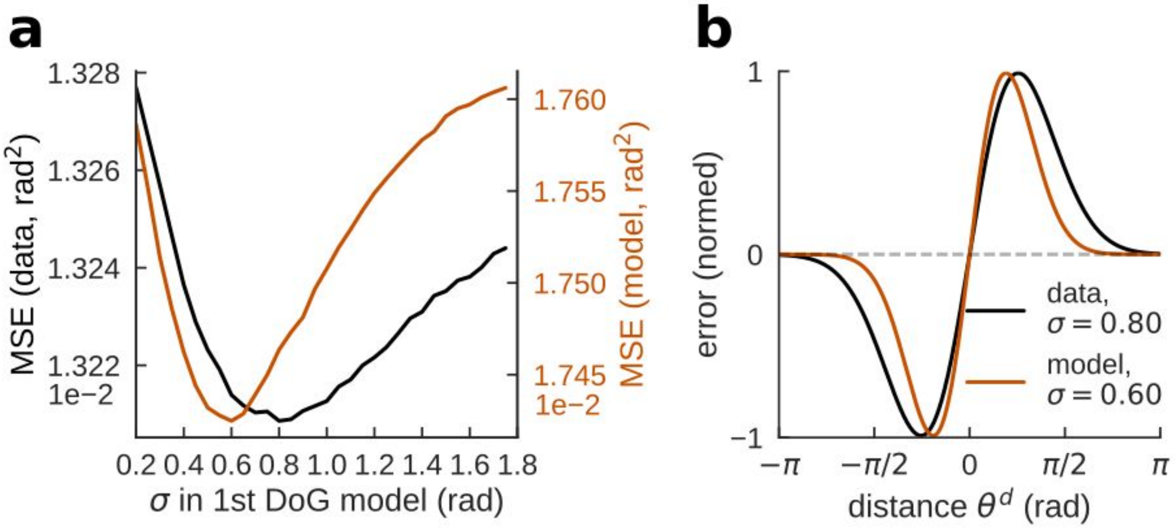

Supplementary Figure 13 | Hyperparameter cross-validation and model selection

a, Mean squared error for stratified hyperparameter optimization using cross-validation (1,000 repetitions, training set size = 0.33 from each subject) for data (black) and for the network model model (orange; cross-validation with 1,000 repetitions, training set size = 0.33 of 21,000 simulated trials with baseline STP and conductance parameters, corresponding to the control condition in Figure 3). Hyperparameters are different values of scale parameter σ (in radians) of the underlying Gaussian with location hyperparameter $\mu = 0$. **b,** Shape of first-derivative-of-Gaussian fits with optimal hyperparameter σ and $\mu = 0$ for data (black) and model (orange). Hyperparameter cross-validation for neural network simulations was carried out for the default parameter values of P, g_{EI} and g_{EE} as reported in Methods. Note that in b, signed previous-current distances are indicated in radians as used in the linear model.

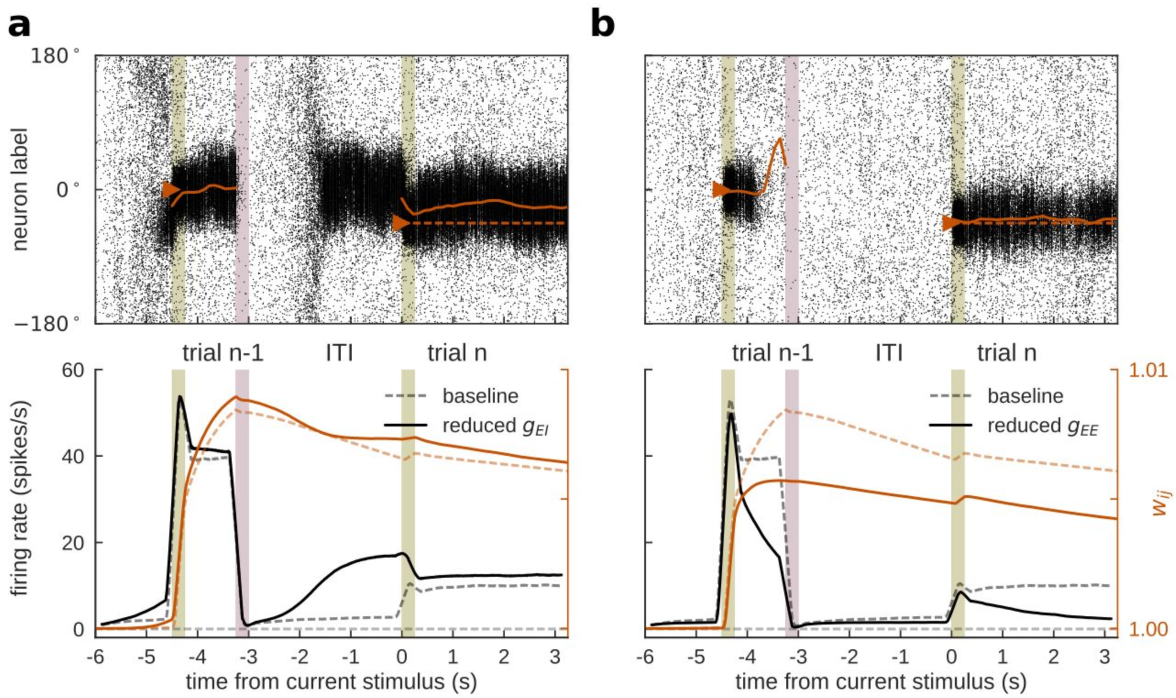

Supplementary Figure 14 | Network behavior for reduced g_{EI} and g_{EE}

a, For reduced g_{EI}, network activity was disinhibited and baseline firing became unstable. Spontaneous activity bumps emerged in the ITI (upper panel), often in neurons that had been active during the previous delay. Lower panel shows firing rates and STP traces at neurons selective to stimuli appearing at 0° for the baseline condition (0% reduction, dashed lines) and the disinhibited condition (1% reduction, solid lines), averaged over 1,000 trials in which the second stimulus appeared at randomized locations. **b,** For reduced g_{EE}, delay firing became unstable and active working memory representations were lost over the delay (upper panel). Lower panel analogous to **a,** for the baseline condition (0% reduction, dashed lines) and the condition of reduced excitation (1% reduction, solid lines). Lower panels were computed as in Figure 2 but including trials for which the same neurons were coactive in the two successive trials. This explains the difference between dashed lines here and in Figure 2b, trial n.

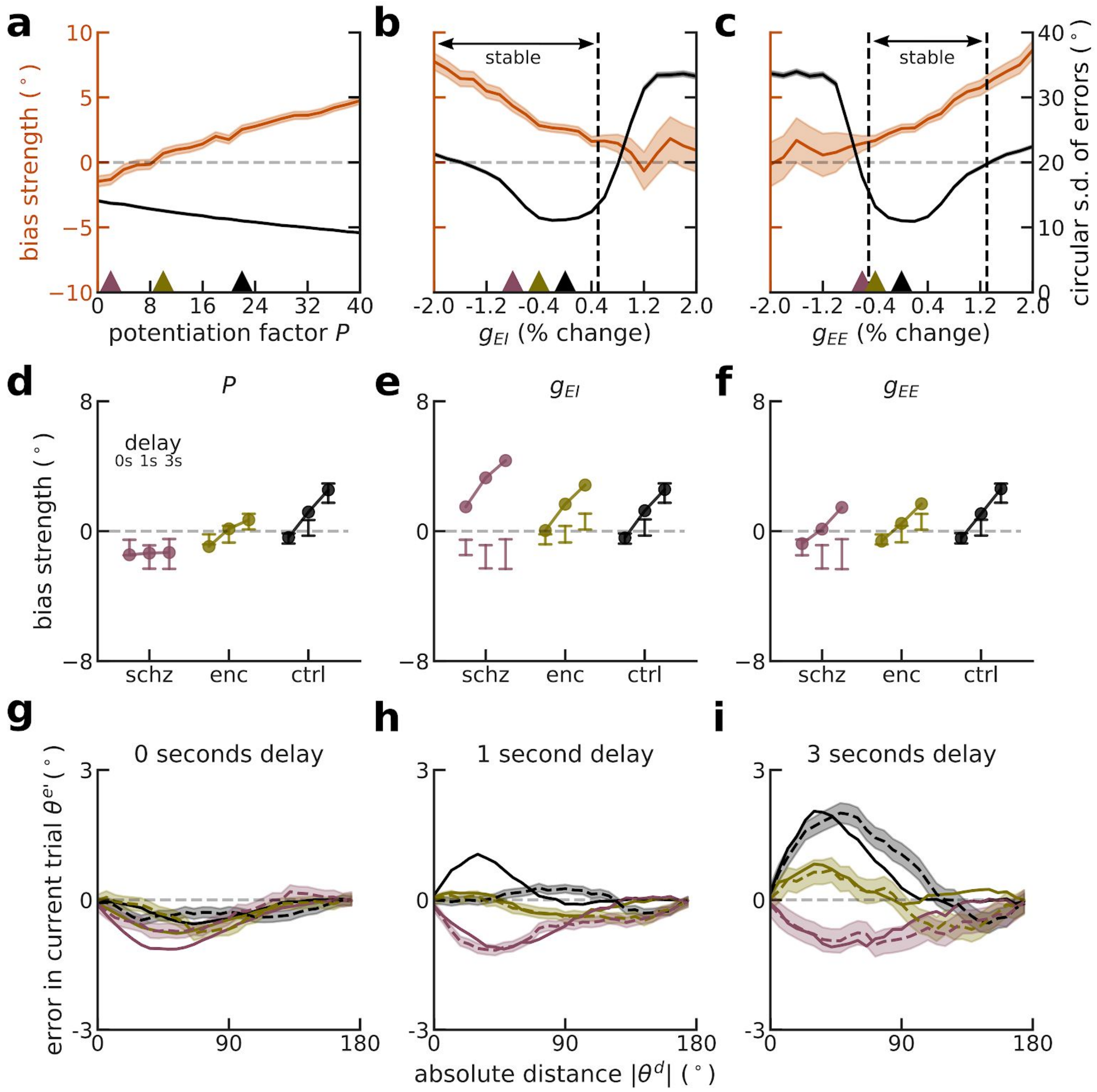

Supplementary Figure 15 | Modeling results are generalizable to networks with STP in both E-E and E-I synapses

Network simulations as in Figure 3, but for STP (and reductions in STP for **a,d,g-i**) in both recurrent excitatory and excitatory-to-inhibitory synapses. **a,b,c,** Serial dependence (orange, bias coefficients from linear model, Methods) and precision (black, circular s.d. of errors) as a function of model parameters in 3 s delay trials (20,000 trials per parameter value). Vertical dashed lines indicate transition to 'unstable' network regimes for which more than 10% of trials were outliers ($|\theta^e| > 57.3°$, i.e. 1 radian). Shading, 95% C.I. for parameter estimates. **a,** Serial dependence decreased gradually when decreasing STP (potentiation factor *P*), while the network remained stable for all simulated values of *P*. Precision changed slightly as a function of STP. **b,** Cortical disinhibition via decreased g_{EI} augmented serial bias while strongly affecting

precision and stability. Note that for the observed parameter values, the network did not enter the disinhibited unstable state (left), unlike in Figure 3. **c,** Lowering recurrent cortical excitation (g_{EE}) led to the opposite pattern, decreasing biases. **d,e,f,** delay dependence of biases for each group, as defined by parameter values in **a,b,c,** (respectively colored triangles). Points depict mean bias strength (over 20,000 trials) for each parameter value. For comparison, error bars indicate 95% CI for bias strength obtained from n = 19 healthy controls (ctrl), n = 17 patients with schizophrenia (schz), and n = 16 patients with anti-NMDAR encephalitis (enc) (reordered from Figure 1g-i). **d,** Lowering STP strength reproduced the experimental data. In **e** and **f,** reduction of NMDAR conductances (g_{EI} or g_{EE}) did not reproduce group and delay dependencies of experimental biases. **g,h,i,** Solid lines, simulated serial dependence by delay length for different values of *P*, indicated by colored triangles in **a** (20,000 trials per potentiation level *P*). Dashed lines with error bars, serial dependence in encephalitis, schizophrenia, and healthy controls. Bias calculated as averaged 'folded' error θ^{e}' for binned absolute previous-current distances θ^{d}. Shading, ± s.e.m. Compare to Figure 3 in the main text for a network with STP (and STP disruptions in patients) only in E-E connections.

Supplementary Tables

	**ctrl (n=19)** mean (s.d.) vs enc/schz	**enc (n=16)** mean (s.d.) vs ctrl/schz	**schz (n=17)** mean (s.d.) vs ctrl/enc	**F-value / Chi-square**	**p-value** (two-tailed)
**age** (years)	22.4 (6.8)	25.5 (6.6)	20.2 (6.1)	2.71	0.08
**gender** (% male)	21.1	12.5	41.2	3.86	0.14
**medication** (% taking antipsychotics)	0.0<br>n.s. / *	25.0<br>n.s. / *	93.3<br>* / *	35.35	2e-8
**medication** (CPZ equivalent, mg day^{-1})	0.0 (0.0)<br>n.s. / *	26.6 (52.7)<br>n.s. / *	370.6 (462.4)<br>* / *	10.44	2e-4
**Global Assessment of Functioning** Scale	86.3 (6.3)<br>* / *	54.9 (13.4)<br>* / n.s.	51.5 (12.7)<br>* / n.s.	54.62	3e-13
PANSS **Positive Symptoms**	7.2 (0.4)<br>n.s. / *	8.1 (1.3)<br>n.s. / *	12.5 (5.1)<br>* / *	15.32	7e-6
PANSS **Negative Symptoms**	7.0 (0.0)<br>* / *	12.9 (5.2)<br>* / *	18.9 (7.4)<br>* / *	24.29	5e-8
PANSS **General Psychopathology**	16.5 (1.2)<br>* / *	26.0 (7.3)<br>* / n.s.	30.2 (9.1)<br>* / n.s.	20.40	4e-7
PANSS **Total Score**	30.7 (1.6)<br>* / *	47.1 (12.7)<br>* / *	61.6 (17.5)<br>* / *	28.70	6e-9
Hamilton **Depression** Rating Scale	0.8 (1.8)<br>* / *	5.4 (4.3)<br>* / n.s.	7.3 (6.1)<br>* / n.s.	10.70	1e-4
Young **Mania** Rating Scale	0.4 (1.1)<br>* / n.s.	4.2 (4.6)<br>* / n.s.	2.6 (2.8)<br>n.s. / n.s.	6.72	0.003

Supplementary Table 1 | Clinical and demographic statistics of the population

Measures: GAF (Global Assessment of Functioning Scale [2]), YMRS (Young Mania Rating Scale [3]), HAM-D (Hamilton Depression Rating Scale [4]), PANSS (Positive and Negative Syndrome Scale [5]) Positive, Negative and General Psychopathology Scale, CPZ equivalent (transversal estimate of antipsychotic medication as chlorpromazine equivalent). The significance of pairwise post-hoc Tukey/Bonferroni-corrected Chi-square tests is reported below group mean and s.d. (n.s. marks non-significant comparisons, and * significant comparisons with FWE = 0.05).

	**baseline (n=14)** mean (s.d.)	**follow-up (n=14)** mean (s.d.)	**t-value / Chi-square**	**p-value** (two-tailed)
**medication** (% taking antipsychotics)	21.4	7.1	0.29	0.59
**medication** (CPZ equivalent, mg day^{-1})	21.4 (48.7)	3.0 (11.2)	1.34	0.20
**Global Assessment of Functioning** Scale	53.4 (12.4)	72.1 (13.7)	-4.69***	4e-4
PANSS **Positive Symptoms**	8.2 (1.4)	8.2 (0.9)	0.00	1.00
PANSS **Negative Symptoms**	12.6 (4.6)	8.6 (2.4)	3.24**	0.006
PANSS **General Psychopathology**	26.3 (7.1)	20.6 (3.2)	2.86*	0.01
PANSS **Total Score**	47.1 (12.1)	37.4 (5.5)	2.85*	0.01
Hamilton **Depression** Rating Scale	5.8 (4.4)	3.4 (4.0)	2.12	0.05
Young **Mania** Rating Scale	4.5 (4.8)	3.1 (2.3)	1.11	0.29

Supplementary Table 2 | Baseline/follow-up comparison of anti-NMDAR encephalitis patients

Measures: GAF (Global Assessment of Functioning Scale [2]), YMRS (Young Mania Rating Scale [3]), HAM-D (Hamilton Depression Rating Scale [4]), PANSS (Positive and Negative Syndrome Scale [5]) Positive, Negative and General Psychopathology Scale, CPZ equivalent (transversal estimate of antipsychotic medication as chlorpromazine equivalent).

References

1. Cicchini, G. M., Anobile, G. & Burr, D. C. Compressive mapping of number to space reflects dynamic encoding mechanisms, not static logarithmic transform. *Proc Natl Acad Sci USA* **111,** 7867–7872 (2014).
2. Bobes, J., Portilla, M. P. G., Bascarán, M. T., Saiz, P. A. & Bousoño, M. *Banco de instrumentos básicos para la práctica de la psiquiatría clínica*. (Psiquiatría Editores S.L., 2004).
3. Colom, F. *et al.* [Spanish version of a scale for the assessment of mania: validity and reliability of the Young Mania Rating Scale]. *Med Clin (Barc)* **119,** 366–371 (2002).
4. Ramos-Brieva, J. A. & Cordero-Villafafila, A. A new validation of the Hamilton Rating Scale for Depression. *J. Psychiatr. Res.* **22,** 21–28 (1988).
5. Kay, S. R., Fiszbein, A., Vital-Herne, M. & Fuentes, L. S. The Positive and Negative Syndrome Scale--Spanish adaptation. *J. Nerv. Ment. Dis.* **178,** 510–517 (1990).
6. Shin, H., Zou, Q. & Ma, W. J. The effects of delay duration on visual working memory for orientation. *J. Vis.* **17,** 10 (2017).
7. Wei, X.-X. & Stocker, A. A. A Bayesian observer model constrained by efficient coding can explain “anti-Bayesian” percepts. *Nat. Neurosci.* **18,** 1509–1517 (2015).
8. Lipinski, J., Simmering, V. R., Johnson, J. S. & Spencer, J. P. The role of experience in location estimation: Target distributions shift location memory biases. *Cognition* **115,** 147–153 (2010).
