# Supplementary material for: Reduced serial dependence suggests deficits in synaptic potentiation in anti-NMDAR encephalitis and schizophrenia
Source: Nat Commun. 2020 Aug 25;11:4250. doi: 10.1038/s41467-020-18033-3 (PMC7447775; doi:10.1038/s41467-020-18033-3)
Supplement: Supplementary file 3 — Reporting Summary [file 41467_2020_18033_MOESM3_ESM.pdf]

nature research

Corresponding author(s): Compte, Albert

Last updated by author(s): Jul 27, 2020

Reporting Summary

Nature Research wishes to improve the reproducibility of the work that we publish. This form provides structure for consistency and transparency in reporting. For further information on Nature Research policies, see Authors & Referees and the Editorial Policy Checklist .

Statistics

For all statistical analyses, confirm that the following items are present in the figure legend, table legend, main text, or Methods section.

n/a	Confirmed	
☐	☒	The exact sample size (n) for each experimental group/condition, given as a discrete number and unit of measurement
☐	☒	A statement on whether measurements were taken from distinct samples or whether the same sample was measured repeatedly
☐	☒	The statistical test(s) used AND whether they are one- or two-sided<br>*Only common tests should be described solely by name; describe more complex techniques in the Methods section.*
☐	☒	A description of all covariates tested
☐	☒	A description of any assumptions or corrections, such as tests of normality and adjustment for multiple comparisons
☐	☒	A full description of the statistical parameters including central tendency (e.g. means) or other basic estimates (e.g. regression coefficient) AND variation (e.g. standard deviation) or associated estimates of uncertainty (e.g. confidence intervals)
☐	☒	For null hypothesis testing, the test statistic (e.g. F, t, r) with confidence intervals, effect sizes, degrees of freedom and P value noted<br>*Give P values as exact values whenever suitable.*
☒	☐	For Bayesian analysis, information on the choice of priors and Markov chain Monte Carlo settings
☐	☒	For hierarchical and complex designs, identification of the appropriate level for tests and full reporting of outcomes
☐	☒	Estimates of effect sizes (e.g. Cohen's d, Pearson's r), indicating how they were calculated

Our web collection on statistics for biologists contains articles on many of the points above.

Software and code

Policy information about availability of computer code

Data collection	behavioral paradigm: custom code written in Psychopy v3.1.5, running in Python 2.7<br>simulations: Brian2 running in Python 3.5
Data analysis	custom code (available at: https://github.com/comptelab/serialNMDA), Python 3.7, R statistics 3.6.3

For manuscripts utilizing custom algorithms or software that are central to the research but not yet described in published literature, software must be made available to editors/reviewers. We strongly encourage code deposition in a community repository (e.g. GitHub). See the Nature Research guidelines for submitting code & software for further information.

Data

Policy information about availability of data

All manuscripts must include a data availability statement. This statement should provide the following information, where applicable:

- Accession codes, unique identifiers, or web links for publicly available datasets
- A list of figures that have associated raw data
- A description of any restrictions on data availability

Behavioral data analyzed in this article are openly available by accessing the github repository: https://github.com/comptelab/serialNMDA

Field-specific reporting

Please select the one below that is the best fit for your research. If you are not sure, read the appropriate sections before making your selection.

☒ Life sciences ☐ Behavioural & social sciences ☐ Ecological, evolutionary & environmental sciences

October 2018

Life sciences study design

All studies must disclose on these points even when the disclosure is negative.

Sample size	No sample size calculation was performed, as the effect we observe in patients has not been reported before. Sample sizes were determined based on the number of geographically accessible (mostly within Spain) anti-NMDAR encephalitis patients that were expected to be identified and eligible to participate in this study in the course of three years. Sample sizes for healthy controls and schizophrenia were determined to match the encephalitis group. In sum, sample sizes were limited by the recruitment rate of anti-NMDAR encephalitis patients, given the low incidence of this disease in the population. The realistic target sample size of 20 participants in each group, however, is in line with previous behavioral studies that have assessed serial dependence in healthy participants (Fischer and Whitney, Nat Neurosci 2014, n=12) and, more importantly, that have compared serial dependence between matched clinical populations (Lieder et al. Nat Neurosci 2019, Experiment 3, dyslexia n=17, autism n=19, healthy n=26), in a design parallel to our study.
Data exclusions	We excluded data from several patients and controls. Note that several subjects were excluded at the beginning of the study, which is due to a change in task protocol that made the data sets from those subjects incompatible with data from later subjects (change of the number of working memory items, contingencies of different delays, and trial randomization).<br>- Healthy controls. Three subjects were excluded due to a change in the experimental protocol (C01,C02,C04). For a fourth subject (C03), we repeated the experiment with the new protocol at a later time point.<br>- Patients with anti-NMDAR encephalitis. For six patients (E01-E07), we excluded the first session due to a change in the experimental protocol and retested all of these subjects several weeks later. Two patients that had been included for other clinical measures were excluded based on their age (E16,E17; 10 and 57 years).<br>- Patients with schizophrenia. Two subjects (S01,S02) were excluded due to a change in behavioral protocol. One subject (S10) aborted the session after less than three blocks and was excluded. One subject had been included for other clinical measures and was excluded based on their age (S20; 49 years).<br>The data exclusions reported above were all established before the analysis of the data.
Replication	In this study, we did not attempt to replicate the presented behavioral findings. This is due to the difficulty of accessing more subjects from the tested patient populations within the temporal limits of the project.
Randomization	Randomization on the level of subjects was not performed. The experimental conditions of our behavioral task were the same for each subject in all different experimental groups, which made randomization unnecessary.<br>On the level of presented stimuli, we fully randomized factors delay (given predefined contingencies) and stimulus position (by randomly sampling from a uniform distribution) between trials.
Blinding	Blinding was not performed at the time of testing or data analysis.<br>During testing, blinding was not possible because patients with anti-NMDAR encephalitis and most patients with schizophrenia were accompanied by family or medical staff. Our behavioral experiment, however, required only a minimum of interaction between the researcher and participants (explanation and task practice, breaks during the task). This means that task performance and other measured parameters should only be minimally influenced by interactions between researcher and participant.<br>At the time of data evaluation, all subjects' behavioral data was evaluated using the same code, including exclusion criteria based on response times, errors, etc. Our analysis is therefore not affected by the lack of blinding.

Reporting for specific materials, systems and methods

We require information from authors about some types of materials, experimental systems and methods used in many studies. Here, indicate whether each material, system or method listed is relevant to your study. If you are not sure if a list item applies to your research, read the appropriate section before selecting a response.

Materials & experimental systems

n/a	Involved in the study	
x		Antibodies
x		Eukaryotic cell lines
x		Palaeontology
x		Animals and other organisms
	x	Human research participants
	x	Clinical data

Methods

n/a	Involved in the study	
x		ChIP-seq
x		Flow cytometry
x		MRI-based neuroimaging

Human research participants

Policy information about studies involving human research participants

Population characteristics	n=16 patients with anti-NMDAR encephalitis (enc)<br>n=17 patients with schizophrenia or schizoaffective disorder (n=12 and n=5, respectively; schz)<br>n=19 neurologically and psychiatrically healthy control participants (ctrl), all with normal or corrected vision.<br><br>For a detailed overview of covariate-relevant demographic and clinical measures, and statistical comparison of group differences, please consult Supplementary Tables 1 and 2.
Recruitment	Patients diagnosed with anti-NMDAR encephalitis were recruited from different centers (n=14 in Spain, n=1 in Germany and n=1 in the United Kingdom) by the time of hospital discharge. They completed the experiment around 5.5 months after disease onset (median, interquartile range i.q.r. = 3.5 months). Based on the recruitment of spontaneously occurring cases in a broad geographical area, we do not suspect specific biases in relevant cognitive dimensions. Socio-economic status (SES) for our encephalitis patients was 47.56 +/- 17.58 (mean +/- std) (Hollingshead, 1957).<br><br>Patients with schizophrenia were recruited from the Barcelona area through the Department of Child and Adolescent Psychiatry, Hospital Clínic, University of Barcelona, Barcelona, Spain. The patients had been or are currently treated in our institution (Hospital Clinic). To facilitate age-matching with the encephalitis group, patients with schizophrenia were recruited without a strict criterion of disease evolution (1-15 years). A self-selection bias of better stabilized, more highly motivated and collaborative patients might exist. The SES for our group of patients with schizophrenia was 26.25 +/- 18.83 (mean +/- std).<br><br>Control participants were recruited from the Barcelona area through advertisements on the project's website and from a database of control participants of previous studies. As this relies on a particular interest in science or volunteering interests, a SES sampling bias could exist, but their SES (54.05 +/- 15.62 (mean+/- std) was not significantly different from anti-NMDAR patients. Of note all groups were approximately matched by age.<br><br>When testing differences in SES between our groups, we found that the schizophrenia group had lower SES than the other two groups and there were no other significant differences. This was as expected from unbiased sampling, as schizophrenia is known to have higher incidence in lower socio-economic strata.
Ethics oversight	Research Ethics Committee of Hospital Clínic

Note that full information on the approval of the study protocol must also be provided in the manuscript.

Clinical data

Policy information about clinical studies

All manuscripts should comply with the ICMJEguidelines for publication of clinical research and a completedCONSORT checklist must be included with all submissions.

Clinical trial registration	*Provide the trial registration number from ClinicalTrials.gov or an equivalent agency.*
Study protocol	*Note where the full trial protocol can be accessed OR if not available, explain why.*
Data collection	Data was collected at the Sleep Unit of the Neurology Department of Hospital Clinic, Barcelona. Participant recruitment started in November 2016, until April 2019. Behavioral testing was performed between January 2017 and June 2019.
Outcomes	We pre-defined two primary variables of interest based on previous experimental and modeling work in healthy controls and patients with schizophrenia, as well as drug studies in healthy participants (administered NMDAR antagonists).<br>The first variable was working memory accuracy, measured as the working memory-delay-dependent standard deviation of response errors. The second variable was serial dependence in working memory, measured as a shift of delay-dependent response distributions towards previous memory items.<br>Secondary measures were general and psychosis-related clinical measures (General Assessment of Functioning Scale, chlorpromazine equivalents of antipsychotic medication, Positive and Negative Psychotic Syndrome Scale, Young Mania Rating Scale, Hamilton Depression Rating Scale). These measures were chosen for a psychiatric description of the sample and the potential relation of primary outcomes with these clinically validated measures.
